# Appropriate or inappropriate ICD shock; what is the post-shock rhythm?

**DOI:** 10.1007/s12471-017-0999-7

**Published:** 2017-05-17

**Authors:** B. M. van Gelder, B. ter Burg, F. A. L. E. Bracke

**Affiliations:** Department of Electrophysiology, Catharina Hospital, Eindhoven, The Netherlands

A 65-year-old male with persistent atrial fibrillation underwent implantation of a dual chamber implantable cardioverter defibrillator (ICD) for primary prevention after previous myocardial infarction with an ejection fraction of 32%. A Boston Scientific 4471 screw-in lead was implanted in the right atrial appendage, and a Medtronic Sprint Quattro Secure 6935 shock lead in the right ventricular (RV) apex. Both leads were subsequently connected to a Medtronic Protecta DR ICD. The intrinsic rhythm at the time of implant was atrial fibrillation with a varying ventricular response below 100 bpm (Fig. 1). Shortly after implant, the patient experienced an ICD shock. Review of the ICD diagnostics showed a sudden onset of a fast rhythm which was diagnosed by the device as ventricular fibrillation according to the fibrillation sense (FS) annotation (Fig. 1), followed by a direct current (DC) shock that terminated the arrhythmia in both the RA electrogram and the RV electrogram (Fig. 2).

**Fig. 1:**
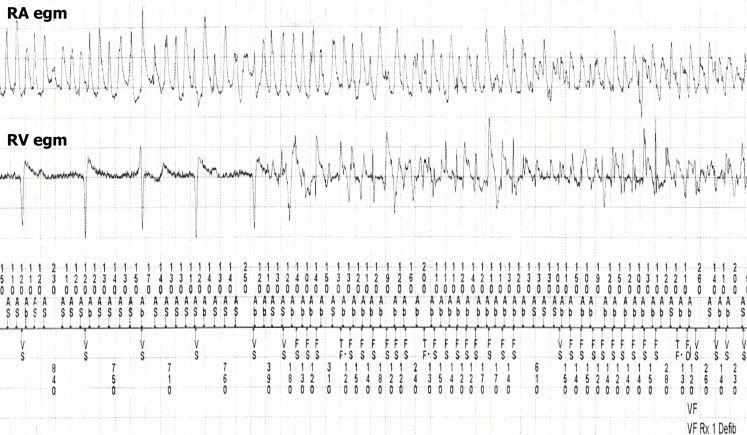
Initial part of the recording shows atrial fibrillation in the atrial electrogram (RA egm) with a varying ventricular response below 100 bpm in the ventricular electrogram (RV egm). There is a sudden change in the RV egm, diagnosed as ventricular fibrillation according to the fibrillation sense (FS) annotation

**Fig. 2:**
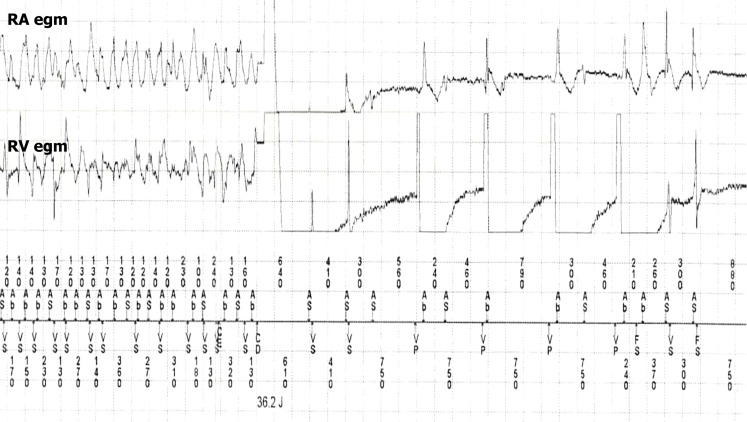
Recording of the ICD shock (*CD* charge delivered) that terminates the arrhythmia in the right atrial (RA) egm and right ventricular (RV) egm

Was this shock appropriate and can you explain the rhythm after the DC shock?

## Answer

You will find the answer elsewhere in this issue.

